# Giant intraosseous schwannoma of scapula: a rare case report and review of the literature

**DOI:** 10.1186/1746-1596-9-31

**Published:** 2014-02-07

**Authors:** Yu Wang Tian, Li Ying Zhang, Zhi Qin Liu

**Affiliations:** 1 Department of Pathology, The Military General Hospital of Beijing PLA, Nanmen Warehouse 5, Dongsishitiao Street, Dongcheng District, Beijing 100700, People’s Republic of China; 2 Department of Image, The Military General Hospital of Beijing PLA, Beijing 100700, People’s Republic of China

**Keywords:** Giant intraosseous schwannoma, Scapula, Surgical treatment

## Abstract

Bony schwannoma is a rare benign tumor derived from schwann cells of nerve fibers in the bone. It accounts for less than 1% of bony benign tumor, and prone to occur in the sacrum and mandible, occurrence in scapula is very rare. Here we report a 42-year-old woman with the chief complaint of pain in the left scapula. Imaging examination showed a giant, irregular, swelling lesion with distinct border involving the left scapula, extending into the left shoulder glenoid and pressing the surrounding soft tissues. Needle biopsy showed that the tumor was composed of spindle cells with S-100 protein positive, mimicking a benign neurogenic tumor. Then a complete excision was performed by removing the tumor and the surrounding tissues including partial left shoulder glenoid. Histologically, Antoni type A areas were the predominant microscopic pattern with occasional alternation by Antoni type B areas. Immunohistochemistry found that the neoplastic cells were scatteredly positive for S-100 protein. All these features suggest a diagnosis of an intraosseous schwannoma of the left scapula. Follow-up of the patient for ten months found no recurrence or sign of other tumors following complete tumor resection without any adjuvant therapy. In conclusion, this case of giant intraosseous schwannoma of the scapula is a rare benign bony tumor, and its diagnosis combined with clinical, imaging and pre-operative needle biopsy is important to guide further therapy, and avoid overtreatment.

**Virtual slides:**

The virtual slides for this article can be found here: http://www.diagnosticpathology.diagnomx.eu/vs/1399352761104042

## Background

Schwannomas (neurilemomas) are benign neurogenic tumors arising from Schwann cells of the peripheral nerves and spinal nerve roots. These tumors have a predilection for the head and neck, the extremities and the posterior mediastinum [1,2]. Schwannoma of the bone is a rare benign tumor accounting for less than 1% of bony benign tumor [3]. Intraosseous schwannoma occurring in sacrum and mandible is higher than in other places [4,5], including the long bones, vertebra, fibula and frontal bone [3,6-8]. Here we report a case of rare giant scapular schwannoma through imaging, morphological and immunohistochemical studies. A ten-month follow-up after the tumor resection without any other adjuvant therapy showed no recurrence or sign of other tumors.

## Case presentation

A 42-year-old female presented to our hospital with left shoulder pain for more than 4 years. The pain usually occurs in the early morning or after exertion, with no obvious effect to physiotherapy. The pain became more severe two weeks ago. There was no trauma history of scapula and she was otherwise healthy.

**Figure 1:**
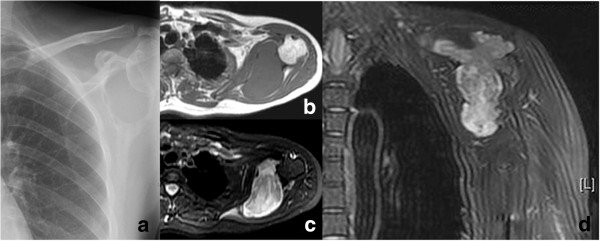
**Imaging inspection. a** Conventional chest X-ray views of an oval mass in the left scapula, with clearer boundary and surrounded by a thin layer of harden zone. **b**, **c** and **d** MRI shows a solitary irregular lobular soft tissue mass measuring about 88 × 80 × 40 mm in the left scapula, the boundary was clear, which showed low intensity signal in T1WI (1b) and high intensity signal in T2WI. (1c) horizontal plane, (1d) coronal plane. The lesion involved the glenoid and entered into surrounding soft tissues, adjacent muscles were compressed and shifted.

Percussion tenderness was elicited on the left shoulder region on physical examination. Laboratory tests revealed no obvious abnormality. Conventional chest X-ray showed an oval low-density region in the left scapula with relatively clear border (Figure 1a). Computerized tomography (CT) conducted in local hospital showed a destruction of the left glenoid and a huge mass in the left scapula. The mass extends into the surrounding soft tissues including the supraspinatus fossa, infraspinatus fossa and subscapularis gap. In addition, magnetic resonance imaging (MRI) identified a solitary irregular lobular mass measuring about 88 × 80 × 40 mm in the left scapula, with less structured contour in the center but clearer boundary, with low to intermediate intensity on T1-weighted images (T1WI) (Figure 1b) and high intensity on T2-weighted images (T2WI) (Figure 1c and d), also with destruction of the left scapula and glenoid. No obvious abnormality was found in the contralateral scapula.

**Figure 2:**
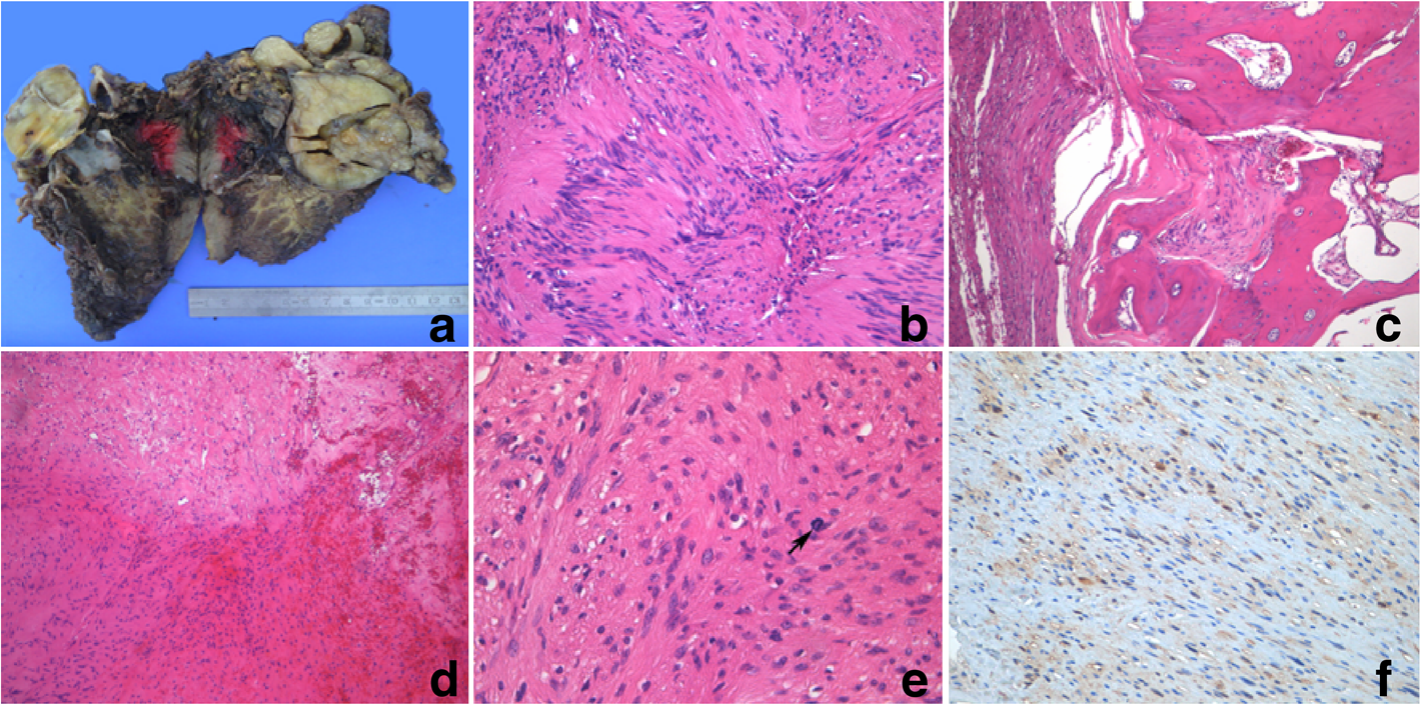
**Gross observation, morphologic features and immunohistochemistry. a** Gross specimen showed the tumor was solid, irregular lobular, the boundary was clear, with two small nodules next the largest one, some part of the tumor connected with the bone. The cut surface was pale yellow, with medium texture. **b** to **e** Hymatoxylin and eosin stain showed the morphologic features of the neoplastic cells. Histological examination of the resection specimen revealed that the greatest portion of the tumour was composed of interlacing bundles of elongated cells with spindle-shaped nuclei (2b). Some tumor tissues located in the bone (2c). Focal area accompanied with bleeding and degeneration (2d). The nuclei were corrugated, atypia was not obvious, enlarged and pleomorphic nuclei could be seen in some region, mitotic figure could be found occasionally (2e, black arrow). (Magnification: 2b and 2e, ×400; 2c and 2d, ×100). **f** Positive IHC signals were visualized with brown yellow color. Cytoplasmic and nuclear immunostaining of neoplastic cells with S-100 protein (Magnification, ×400).

Needle biopsy showed that the tumor was composed of spindle cells, S-100 protein was positive with immunohistochemistry. Therefore, it was considered a neurogenic benign tumor, specifically schwannoma. Then she received a complete left scapular tumor resection. During the surgery, a dark red nodular mass was seen below the left shoulder joint. The proximal cartilage of the glenoid was fragmented and the joint capsule was cut, there was no effusion in the joint cavity. The nodular mass grew in a dumbbell-shaped configuration, with one side in the soft tissue and the other side entered into the joint surface. There were two small nodules around the big one, and the borders of them were also clear. The tumor along with part of the scapula and some surrounding soft tissues were surgically removed and refilled with artificial bone cement. The gross specimen showed a solid lobular tumor with clear boundary, partly located within the bone. The largest nodule measured 6.0 × 5.0 × 4.5 cm, and the two smaller nodules next the largest one measured 3.5 and 2.0 cm in diameter respectively. The cut surface of the tumor was yellowish white, smooth, solid with medium texture, local edge was a little hard (Figure 2a).

Specimens were fixed in 10% neutral formalin, dehydration, paraffin-embedded. The hard areas were cut into sections (4 μm thick) after decalcification. HE and immunohistochemical stains were performed. The primary antibodies included S-100, SMA, Desmin, CD117, CD34 and Ki-67. All primary antibodies were mouse anti-human monoclonal antibodies, dilated 1:100 (Zymed Laboratories, South San Francisco, CA). A DAB (3, 3′-diamino-benzidine-tetrahydrochloride) substrate-chromogen kit (Zymed Laboratories, South San Francisco, CA) was used to visualize signals. All sections were counterstained with hematoxylin.

### Histopathology

Microscopic examination showed that the tumor mass was composed of spindle cells with twisted nuclei and the low-density areas (Antoni Type B) staggered with high density areas (Antoni Type A). The nuclei were palisading arrangement and formed the "Verocay bodies". The morphological features of the tumor mass were similar to that of the needle biopsy specimen (Figure 2b). Some tumor tissues were founded in the bone (Figure 2c). Focal areas showed bleeding and degeneration (Figure 2d). Atypical nuclei were not obvious in most of the region, but enlarged and pleomorphic nuclei could be seen in small areas of the region. Mitotic figures could be found occasionally (Figure 2e).

Expression of S-100 was found in some of the cytoplasm and nuclei of the spindle neoplastic cells (Figure 2f). SMA, Desmin and CD117 were all negative. CD34 was positive only for the vascular endothelial cells. The Ki-67 proliferation index was less than 2%.

The patient did not receive any adjuvant therapy after the tumor resection. Until the time of writing, she had been followed up for 10 months after the tumor resection, no sign of recurrence or other tumor was noticed.

## Discussion

Schwannoma is a benign tumor originating in schwann cells of the nerve fibers. It usually occurs in the head and neck. Intraosseous schwannoma is rare accounting for <1% of primary benign bone tumors [3]. Besides of occurring in the sacrum and mandible, intraosseous schwannoma also occurrs in the long bones, vertebra, fibula and frontal bone, etc. The symptoms are mild, and the tumor is usually symptom free until the tumor becomes larger with visible pain and/or swelling. X-ray usually shows a benign bone tumor mass. MRI is particularly helpful in pre-operative diagnosis as it shows the internal texture characteristics of the encapsulated mass. But this method is not applicable to all schwannoma.

The histopathological features of intraosseous schwannoma are similar to soft tissue schwannoma. The tumor cells have spindle, fences-like arrangement cells and may have alternating Antoni type A and B areas. The nuclei can be pleomorphic, but mitotic figure is rare. Large schwannomas (> 8 cm) often undergo cystic degeneration due to haemorrhage or necrosis [9,10]. Neither tumor size nor mitotic figure has been found to reflect malignant behavior [11].

In this case the patient appeared pain of left scapula for more than four years, combined with X-ray, CT and MRI inspections and histologically showing manifest Antoni type A and B patterns. Most of the nuclei were moderate, and immunohistochemically some of the neoplastic cells were positive for S-100 protein, the Ki-67 proliferation index was very low. The final diagnosis of giant scapular schwannoma was made, which was consistent with the needle biopsy results. Sometimes, the imaging appears features of benign tumor, the histological features also show the morphology of schwannoma, so both the imaging and histology show benign tumor features [12]. However, although with advanced imaging techniques even with fine needle biopsy, sometimes schwannoma still will be misdiagnosed pre-operatively [13]. Because of the rarity of these intraosseous schwannoma, it is important that the diagnosis is made pre-operatively by the pathologist examining the needle biopsies and the radiologist interpreting the scans. The diagnosis of this benign tumor may influence further treatment by limiting surgical invasiveness and avoiding unnecessary adjuvant therapy.

Clinically it is often difficult to judge whether it is the outside soft tissue schwannoma involving the bone or intraosseous schwannoma involving the surrounding soft tissues, as they have similar histopathological morphology. Schwannomas can involve bone by three mechanisms: (1) tumors may arise centrally within bones; (2) tumors can arise within the nutrient canal and grow in a dumbbell-shaped configuration, enlarging the canal, or (3) extraosseous tumors can cause secondary erosion of bone [14]. On the base of imaging examination, intraoperative findings of dumbbell-shaped tumor in and outside the scapular glenoid and gross examination, this case demonstrates an example of intraosseous schwannoma.

For differential diagnosis, neurofibroma, malignant peripheral nerve sheath tumor (MPNST), fibrous histiocytoma, and nonossifying fibroma should be distinguished. Neurofibroma may be a manifestation of neurofibromatosis type 1 (NF-1) and the probability of recurrence in neurofibroma is higher than schwannoma [15]. Neurofibroma has the potential for malignant transformation and about 15–16% of patients with neurofibromatosis present with malignant transformation [16]. Neurofibromas lack the thick collagenous capsule of schwannomas. MRI is particularly helpful in showing the internal characteristics of the encapsulated mass. Neurofibromas also lack the Antoni type A and B patterns and "Verocay bodies" which are ususlly apparent in schwannomas. MPNST represents 5-10% of all soft tissue sarcomas and is often associated with NF-1 [17]. However, there was case reported that MPNST had no relationship with NF-1 [18]. MPNST occurs in man more than in woman and occurrences in the skin, head and neck, mediastinal and retroperitoneal are higher than in other places. It was even reported in the ulna of a dog as a model to study human [19]. MPNST usually grows rapidly. Histologically, MPNST usually are comprised of infiltrative, dense and relatively uniform spindle or oval neoplastic cells. Pleomorphic and atypical neoplastic cells are obvious, and mitotic figure and necrosis could be found easily. The Ki-67 proliferation index in MPNST is much higher than in benign schwannoma. Fibrous histiocytoma usually is composed of spindle cells, multinucleated osteoclast-like giant cells, foamy cells and chronic inflammatory cells, often along with interstitial hemorrhage and hemosiderin. Pelvis, especially the ilium, is the most common site of fibrous histiocytoma. It also occurs in the backbone of tibia and fibula, but it requires a combination of clinical, radiological examnation to exclude other bone tumors such as giant cell tumor of bone, nonossifying fibroma, etc. to make a fibrous histiocytoma diagnosis. Nonossifying fibroma often occurs in young people less than 20 years-old, clinically with less pain. It usually occurs in the metaphyseal of long bone. Radiographically it has bubble-type sclerosis edge. The diagnosis of nonossifying fibroma mainly relies on clinical symptoms and imaging which is similar to that of fibrous histiocytoma.

Almost all the intraosseous schwannomas behave in a benign manner, similar to extraosseous soft tissue schwannomas. Primary schwannomas of the bone can be successfully treated by local complete excision alone [3]. Some schwannomas would malignant change [20,21] and mandibular malignant schwannoma has been reported [22]. Through imaging, morphology and immunohistochemistry, we diagnosed that this case is a rare giant benign intraosseus schwannoma occurring in the scapula. In view of the huge tumor size, accompanied with hemorrhage and degeneration, focal areas appeared pleomorphic, atypical and mitotic neoplatic cells, low malignant potential could not be completely ruled out, although the follow-up showed no reccurrence or sign of other tumors in the patient for 10 months after complete tumor resection. It needs a long-term follow-up to observe the biological behavior of this intraosseous schwannoma.

## Conclusion

In conclusion, we report here a rare case of giant intraosseous schwannoma in the left scapula on the basis of imaging, histopathological pattern and immunohistochemical expression of S-100 protein. The histological features of this tumour suggest that this intraosseous schwannoma was a benign tumor. The use of immunohistochemistry may be helpful in distinguishing this type of neoplasm from other spindle cell tumors with similar morphology. Finally, the diagnosis was made pre-operatively from clinical and radiological findings, and needle biopsy results, that was beneficial to the patient for avoiding unnecessary adjuvant therapy.

## Consent

Written informed consent was obtained from the patient for publication of this Case Report and accompanying images. A copy of the written consent is available for review by the Editor-in-Chief of this journal.

## Abbreviations

T1WI: T1-weighted images; T2WI: T2-weighted images; MRI: Magnetic resonance imaging; MPNST: Malignant peripheral nerve sheath tumor.

## Competing interests

The authors declare that they have no competing interests.

## Authors’ contributions

YWT and LYZ collected the data and drafted the manuscript. YWT carried out the immunohistochemistry staining. LYZ made the final diagnosis of this disease. ZQL provided the imaging data and diagnosis. All authors have read and approved the final manuscript.
